# Growth and Maturation in Development: A Fly’s Perspective

**DOI:** 10.3390/ijms21041260

**Published:** 2020-02-13

**Authors:** Renald Delanoue, Nuria M. Romero

**Affiliations:** 1 University Côte d’Azur, CNRS, Inserm, Institute of Biology Valrose, Parc Valrose, 06108 Nice, France; 2 Universitey Côte d’Azur, INRA, CNRS, Institut Sophia Agrobiotech, 06900 Sophia Antipolis, France

**Keywords:** growth, maturation, IIS/IGF, steroid hormones, AstA/Kiss, *Drosophila*

## Abstract

In mammals like humans, adult fitness is improved due to resource allocation, investing energy in the developmental growth process during the juvenile period, and in reproduction at the adult stage. Therefore, the attainment of their target body height/size co-occurs with the acquisition of maturation, implying a need for coordination between mechanisms that regulate organismal growth and maturation timing. Insects like *Drosophila melanogaster* also define their adult body size by the end of the juvenile larval period. Recent studies in the fly have shown evolutionary conservation of the regulatory pathways controlling growth and maturation, suggesting the existence of common coordinator mechanisms between them. In this review, we will present an overview of the significant advancements in the coordination mechanisms ensuring developmental robustness in *Drosophila*. We will include (i) the characterization of feedback mechanisms between maturation and growth hormones, (ii) the recognition of a relaxin-like peptide Dilp8 as a central processor coordinating juvenile regeneration and time of maturation, and (iii) the identification of a novel coordinator mechanism involving the AstA/KISS system.

## 1. Steroid Hormones Promote the Juvenile to Adult Maturation Transition

**Figure 1:**
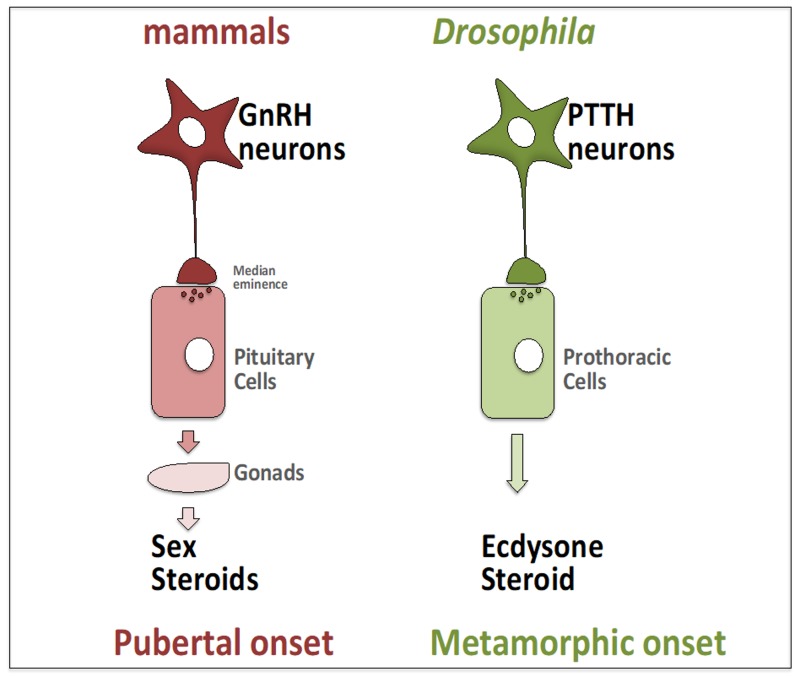
The brain instructs steroid hormone/s production to trigger the juvenile to adult maturation transition in mammals and *Drosophila*.

In several species, the right time to initiate the juvenile-to-adult transition is determined by an increase of steroid hormones production and secretion instructed by the brain. For instance, in mammals, the pubertal onset is marked by the rise of gonadal steroid hormone production as a consequence of the activation of the hypothalamic-pituitary-gonadal (HPG) axis [1] (Figure 1). Pulsatile secretion of hypothalamic neuropeptide gonadotropin-releasing hormone (GnRH) induces the production of luteinizing hormone (LH) and follicle-stimulating hormone (FSH) in the pituitary gland. LH and FSH, through the circulatory system, reach the gonads to stimulate sex steroid hormones production. Consequently, increasing levels of circulating sex steroids promote the pubertal process to acquire adult morphology and physiology [2,3,4]. 

The juvenile to adult developmental transition in *Drosophila* is also triggered by increased levels of the only insect steroid hormone, ecdysone. The production of ecdysone is stimulated by an neuropeptide named prothoracicotropic hormone (PTTH) [5,6]. PTTH is produced by a pair of bilateral neurons that project their axons into the prothoracic gland (PG). PTTH neuropeptide is secreted in the PG to stimulate ecdysone production [7,8]. Consequently, ecdysone induces the developmental transition by orchestrating the metamorphic changes in growth, maturation, and morphology [9]. PTTH neurons (PTTHn) are of cardinal importance to trigger juvenile to adult maturation transition throught a complete metamorphosis. Indeed, PTTH neuron ablation or PTTH mutant flies show a significant delay in the onset of metamorphosis associated with decreased survival rates [7,8,10]. Therefore, mammalian GnRH and insect PTTH play similar crucial roles in gating the beginning of the juvenile to adult maturation transition [11]. Moreover, the PTTH neurons in *Drosophila* and hypothalamic GnRH neurons in mammals control the release of hormones at the PG or pituitary gland through axonal communication (Figure 1).

In most animal species, transition into adulthood only occurs after the appropriate amount of growth has been completed during juvenile stages [12,13,14]. This is essential for emergence of healthy and reproductive adults with correct body size and proportions. It implies that the systems controlling juvenile growth and developmental timing of maturation have to be intimately coupled. Animal genetics largely determines its adult final body size. Nevertheless, plasticity has been introduced in the system to allow adaptation of developing animals to hostile and ever-changing environments [15]. For instance, an extension in the juvenile growth period occurs as a result of food scarcity or tissue injuries [16,17]. Therefore, sensor mechanisms detecting lack of nutrients, loss of tissue mass, or excessive cell death exist to induce a plastic response maintaining the homeostasis of the developing organs. Such mechanisms are essential to adjust the duration of juvenile growth, activate cell proliferation, and restore organ size and functions before the onset of maturation.

## 2. The Insulin/IGF Signal Controls Juvenile Growth

The growth hormone (GH)/insulin-like growth factors (IGF) axis is one of the key effector pathways in growth control. It is believed that most of the growth-promoting effects of GH is mediated by the IGFs [18], suggesting that regulation of insulin/IGFs production is central in coupling growth with maturation. For the sake of simplicity, it is often proposed that insulin regulates metabolism, while IGF is involved in systemic growth. However, despite these distinct developmental roles, insulin and IGFs show overlap in these functions. 

Over the last 15 years, studies in *Drosophila melanogaster* larvae have been particularly significant in understanding the physiology of juvenile growth control in response to environmental stress, such as nutritional variations. Studies performed in this model were crucial in demonstrating that inter-organ communication is predominant in these processes. In flies, the key regulator responding to the nutritional status remains the insulin/IGF signaling (IIS), the evolutionarily conserved signaling pathway. Under feeding conditions, *Drosophila* insulin-like peptides (DILPs) are secreted into the circulatory system. DILPs carry both functions, metabolic and growth regulators, by activating the ubiquitously expressed receptor, insulin receptor (InR). Subsequent activation of downstream components of the insulin pathway positively act on all the mechanisms required for cell and tissue growth, such as cellular uptake of nutrients and stimulation of protein and lipid synthesis. The *Drosophila* genome encodes eight insulin-like peptides (*Drosophila ILP*, *dilp1–dilp8*). DILP6, produced by the fat body (functionally related to the vertebrate liver and white adipose) and glial cells, have IGF-like functions [19,20]. DILP8, provided by growing tissue, share similarities with Relaxin [21,22]. Additionally, DILP1, 2, 3, and 5 secreted from bilateral clusters of neurosecretory cells located in the larval brain (the insulin producing cells or IPCs) are at the central core of glycemia and body growth regulation [23]. Interestingly, DILPs secretion from IPCs is not cell-autonomously regulated but relies on humoral signals emitted by the fat body (FB) [24,25]. Indeed, amino acid shortage or inhibition of the TOR-signaling pathway, in FB cells, is sufficient to induce DILPs retention in these neurons [25]. Original findings showed that humoral signals, named FB-derived signals (FDS), are not metabolites since carbohydrate, lipids, or amino acids were unable to promote DILPs secretion. Recent publications lead to an elaborate regulation scheme where the FB produces specific secreted factors (FDS) in response to distinct nutritional cues like sugar, fat, or protein (amino acids). 

The requirement of cytokines in this humoral inter-organ communication was among the first tested candidates leading to the identification of Upd2 as a nutrient-regulated signal from the FB. Its FB production only decreases upon prolonged starvation and is most likely responding to dietary fats and sugars. Upd2 activates Janus kinase (JAK)/signal transducer and activator of transcription (STAT) signaling in a group of GABAergic neurons, relieving inhibitory effects on the IPCs, which result in DILPs secretion into the hemolymph to promote systemic growth and fat storage [26]. Interestingly, Upd proteins show similarities with Leptin [27], and FB-specific expression of human Leptin rescues *upd2* mutant growth phenotype and stimulates DILPs production [26]. Transcriptional analysis revealed the existence of another FDS, a peptide found in arthropods, CCHa2, mostly responsive to dietary glucose. Its receptor CCHa2-R is highly expressed in the IPCs, and its loss of function leads to changes in DILP2 secretion and DILP5 expression/secretion [28]. The CCHa2-R receptor belongs to the same phylogenetic group as the BRS-3 orphan receptor. BRS-3 is expressed in mouse and human pancreatic β-cells, and its activation by an agonist stimulates insulin secretion [29].

Four FDS specifically responding to nutritional amino acids (protein) have been identified with different properties and modes of action. First, a genetic screen led to the identification of the couple receptor/ligand, Methuselah/Stunted (Mth/Sun), which possesses unique features [30]. The ligand Sun is the ε subunit of the mitochondrial F1F0-ATP synthase complex V, required for the ATP synthase. Surprisingly, it is also detected circulating in the hemolymph, and its levels vary according to nutritive amino acid but not sugar. Hence, Sun has an insulinotropic activity requiring Mth function in the IPCs, independently of its mitochondrial localization [30]. Subsequently, two other FDS were recently identified, the growth blocking peptides, GBP1, and GBP2 (GBP1/2). These peptides are atypical EGF ligands produced by fat body cells, and their expression is regulated by amino acid intake. Lack of GBP1/2 from fat cells lowers DILPs secretion by the IPCs limiting systemic IIS and body growth. Conversely, direct incubation of GBP1/2 on starved brains triggers DILPs secretion [31]. GBP1 and GBP2 have been detected circulating in the hemolymph and signals to the IPC through a double inhibition neuronal relay [32]. Finally, along with insulinotropic factors responsive to amino acids, one negative regulator of DILPs production has been identified. The TNF homolog Eiger, produced by the fat body, remotely and negatively regulates DILPs production by the IPCs. Its release from the fat body relies on the convertase enzyme TACE, and TACE expression is induced under a low protein diet. In the hemolymph, the circulating Eiger remotely acts on the IPCs through the TNF receptor, Grindelwald, and the downstream JNK signaling to regulate *dilp2* and *dilp5* transcriptions. Interestingly, this regulation is not required for DILPs retention upon acute protein starvation but is part of an adaptive mechanism required to adjust *dilps* transcription under chronic dietary amino acid shortage [33]. This mechanism has been conserved through evolution since TNF-α inhibits *INSULIN1* and *INSULIN2* transcription in insulinoma-derived MIN6 cells and in mouse pancreatic islets [33].

Besides nutritional adaptation, a variety of other environmental factors control juvenile growth and influence larval to pupal transition. For instance, IPCs are directly innervated by cold-sensing neurons. Under cold temperature, these neurons stimulate the expression and the release of the DILPs [34]. This mechanism is proposed to sustain metabolism and larval growth without increased food consumption in adverse conditions. A recent report shows that hypoxia slows larval growth and delays development. Interestingly, fat cells are the primary sensor of oxygen availability and inter-organ communication is at play in these regulations. It involves the homolog of the Hypoxia-inducible factor 1 alpha (HIF-1α), the transcription factor Similar (Sima), and the homolog of HIF-1α prolyl hydroxylase (Hph) named fatiga (Fga) in the fat body and a remote control of DILPs production in the IPCs [35]. In addition, it was shown that the TOR signaling in the fat body contributes the hypoxic response to modify juvenile growth and developmental transitions when oxygen levels are low [36]. The presence of microbiota is another factor important for sustaining optimal larval development and conferring an adaptation to the environment, probably by providing a better use of nutrients by the larvae [37].

## 3. Feedback Mechanisms between Maturation and Growth Hormones

**Figure 2:**
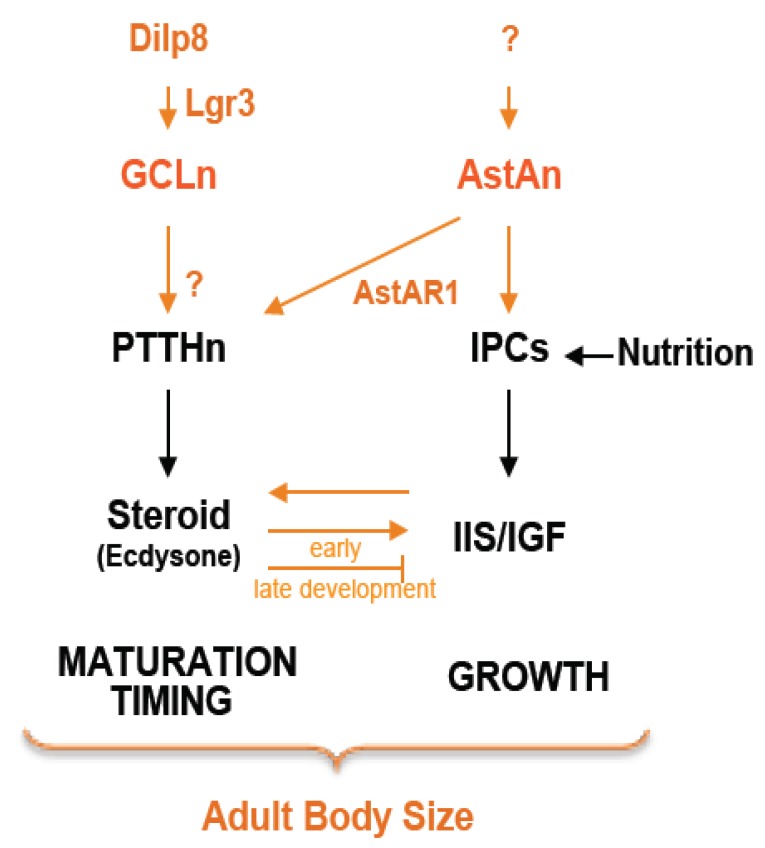
Growth and maturation coordinator mechanisms.

During the juvenile growth period, an intricate cross-regulation exists between the IIS and ecdysone signaling. Indeed, while high levels of ecdysone at maturation time appear to impair tissue growth [38], the IIS pathway stimulates steroid production during the juvenile growth period. IIS regulation on ecdysone production is an early event that leads to a commitment to maturation [39] (Figure 2). Therefore, the level of IIS activation on the PG during development determines the timing of maturation. For instance, high activation of the IIS in PG led to increased steroid circulation levels and precocious timing of metamorphosis since it regulates the expression of steroid biosynthetic enzymes [40,41]. There is some insight into the molecular actors involved in this regulation. Foxo, the transcriptional effector of IIS, represses ecdysone production by binding to Ultraspiracle (Usp), the dimerization partner of ecdysone receptor (EcR). Increasing IIS phosphorylates Foxo and promotes its dissociation from Usp, relieving the inhibition of ecdysone synthesis. This regulation would be an early event leading to a commitment to maturation [39]. Additionally, the regulation of ecdysone production by insulin signaling also relies on the repression of microRNA *bantam*, which has a well-established role in cell-autonomous growth [42].

Several additional signaling mechanisms have been shown to link the external environment with ecdysone production, suggesting that a convergence of inputs is required to adjust juvenile-to-adult transition. For instance, within the ecdysone producing cells, the TOR (target of rapamycin) and TGF β/activin signaling ensures that nutritional and developmental inputs are synchronized [43,44]. Other reports show a requirement of autophagy for coupling developmental timing with nutritional status. This process depends on the growth pathway Hippo/Yki, and one of its target, the microRNA *Bantam*, which regulates downstream effectors such as EcR signaling and the TOR pathway [45,46,47]. Additionally, a subgroup of serotonergic neurons innervates and stimulates the PG in response to rich food quality [48]. Finally, the expression of a predicted amino acid transporter gene (*sobremesa*) in the glial cells is somehow essential for the proper timing of development [49].

Steroid hormones have a dual impact on cell proliferation and growth, and the underlying mechanisms are well documented in *Drosophila* larvae. Once again, inter-organ communication is central in these regulations. During the juvenile growth period, ecdysone and its receptor, EcR appeared to be necessary for cell proliferation [8,50,51]. Indeed, in growing tissues, EcR inactivation or impaired ecdysone synthesis led to reduced proliferation rate [8,51]. However, at the end of the juvenile growth period, rising levels of circulating ecdysone have a negative impact of systemic growth [38]. At least three fat-body dependent mechanisms ensure the ecdysone inhibitory effect. First, fat body activation of E/EcR represses the expression of the oncogene Myc and, therefore, ribosomal RNA synthesis and ribosomal quantities [50]. This finding suggests that ecdysone signaling reduces translational activity in the fat body, probably to restrict the availability of trophic factors required for systemic growth when maturation takes place. Second, an increase in ecdysone production is observed when nutritional restriction is experienced at late juvenile development. This increased circulating ecdysone level activates EcR in the fat body and upregulates the production of the IGF-binding partner (IGF-BP) ImpL2. Consequently, Imp-L2 binds to and inactivates the circulating DILPs attenuating peripheral IIS and body growth [52]. Third, by repressing miRNA *mir8*, the rise of ecdysone titers upregulates the mir8 target U-shaped which is a negative regulator of insulin signaling [53]. Altogether, these data indicate that basal steroid ecdysone levels during development are required for proliferative tissue growth. However, later in development, higher ecdysone levels synchronously arrest systemic growth before metamorphosis and this relies on centralized regulations taking place in the fat body.

## 4. The Relaxin-Like System, DILP8/Lgr3, Coordinates Juvenile Regeneration, and Time of Maturation

As previously mentioned, tissue injuries perturb organ development. To preserve the integrity of developing organs, animals have detection mechanisms in charge of both delaying maturation and stimulating cell proliferation. These events are important for restoring organ size and function. Moreover, animals with determinate growth show a decline in injury-induced regeneration after juvenile to adult transition. This reveals a requirement to stop the maturation process and preserve the regenerative capacity until the damaged tissue is repaired. Evidence that such coordination mechanisms exist comes from experiments dating back to the 1970s showing that *Drosophila* larvae delayed the onset of metamorphosis upon damage induced to growing organs [54]. Subsequent findings demonstrated that the delay occurs by PTTH-PG axis inhibition, which is mediated by retinoids [55] and *Drosophila* insulin-like peptide 8 (DILP8) [21,22] (Figure 2).

Two groups independently identified DILP8 as a divergent insulin/relaxin-like peptide linking juvenile organ growth to developmental timing [21,22]. DILP8 secreted by damaged growing tissue activates the orphan leucine-rich repeat-containing G-protein-coupled receptor 3 (Lgr3) expressed by two pairs of neurons located in the central brain, the growth coordinating Lgr3 neurons (GCLn) [56,57,58]. GCLn signals to PTTH neurons by an unknown mechanism to inhibit ecdysone production and the onset of juvenile to adult transition during the regeneration period [56,57,58]. Thus, the DILP8-Lgr3 signaling couples information from the damaged tissue with the maturation PTTHn-PG axis to maintain homeostasis of tissue size (Figure 2). 

Additionally, Lgr3 is required in the PG to slow down the growth of undamaged tissue via activation of nitric oxide synthetase (NOS) [59]. The reduction in ecdysone leads to a delay in the juvenile to adult transition concomitant with a reduction in the growth rate of non-affected tissues. Further studies are required to explain how regeneration of damaged tissue is promoted under these reduced ecdysone conditions to mediate organ growth coordination. 

The finding that DILP8 released by tissue injury negatively regulates the PTTHn-PG axis suggests that under physiological conditions the DILP8/LGC system may define maturation timing by removing its inhibitory effect once the organism has grown enough. Accordingly, *dilp8* is expressed during the growing juvenile development, and its expression significantly drops just before the onset of maturation [21]. Nevertheless, *dilp8* loss of function does not cause larvae to undergo earlier metamorphosis [22], implying that the absence of DILP8 is a permissive signal rather than an instructive one for maturation. It is also plausible that the DILP8/Lgr3 relaxin-system plays a key role in adjusting premetamorphic growth and maturation programs only upon growth perturbations such as injury or tumor, an essential plastic response to a hostile environment.

## 5. DILP8/GCL System Determines Bilateral Organ Size Symmetry Ensuring Developmental Stability 

The vast majority of animal phyla display proportional and bilateral symmetric bodies like Chordata, Annelida, Arthropoda, Platyzoa, Nematoda, and most Mollusca. There are many advantages of a bilaterally symmetric body such as balance improvement, visual perception, directional movement, etc. To achieve individuals of correct symmetry and proportions, mechanisms allowing inter-organ growth coordination are indispensable. Their existence has been revealed in several organisms by inducing a local growth perturbation. For instance, in mice limb cartilage, unilateral growth inhibition reduces contralateral bone growth, maintaining left-right bone symmetry [60]. In *Drosophila*, artificially slowing down the growth of a subset of tissues reduces the growth rate of unperturbed tissue contributing to the maintenance of a proportional body [16]. By using the fly’s growth-impair model, the previously described relaxin-like peptide DILP8 and the steroid hormone ecdysone were identified as the leading players for the intra-organ size coordination mechanism [61]. RNAi-mediated inhibition of *dilp8* in the slow-growing tissue, or feeding animals with 20E, the active form of ecdysone, fully rescued the systemic growth inhibition of undamaged organs [61]. 

DILP8 coordinates inter-organ size also in the absence of tissue-growth perturbation, reflecting a physiological role for DILP8 in establishing developmental stability. Indeed, *dilp8* or *lgr3* mutant flies display imperfect bilateral symmetry showing intra-individual size variation between the left and right wings [22]. DILP8 stabilizes the size between the distinct body parts by signaling towards Lgr3-GCL neurons, which control the production of the steroid hormone, ecdysone, through PTTH neurons. Silencing *lgr3* in those neurons induced a similar level of organ asymmetry as that observed in *dilp8* or *lgr3* mutants [56,57,58]. The mechanisms by which DILP8 is cell-autonomously upregulated to kick off the inter-organ coordination mechanism depends on the model. Whereas, c-Jun N-terminal kinase (JNK) pathway, Notch signaling, or stress-response transcription factor Xrp1 is required in the case of artificially impaired growth [16,22,57,61,62], the growth regulator Hippo/Yki pathway directly regulates *dilp8* expression under physiological conditions [63]. The current model establishes that JNK, Xrp1, or hippo pathway connects organ growth status to the DILP8/GCL system and that DILP8 activation of GCL neurons leads to PTTHn inhibition to slow down ecdysone production [21,22,56,57,58,61,63]. Thus, reduced steroid levels are responsible for the inter-organ final size assessment mechanism that maintains animal proportions and bilateral symmetry.

Another intriguing finding is that while perturbed tissue growth activation of DILP8/GCL delays the onset of metamorphosis, the physiological activation of the DILP8/GCL signaling that coordinates inter-organ size occurs does not [21,22]. Then, there are still some open questions: why does the physiological activation of the DILP8/GCL not delay juvenile to adult transition? When does the DILP8 size adjustment mechanism take place? It would be reasonable that this mechanism occurs during the growth period, and more rounds of cell proliferation still happen at the beginning of metamorphosis. Thus, it is plausible that the DILP8 physiological size adjustment mechanism occurs right after the onset of metamorphosis, explaining why its physiological activation does not affect maturation timing. However, it is not possible to rule out that during the larval growth period, tiny changes in ecdysone levels might contribute to the intra-organ coordination mechanism without affecting the time of metamorphic onset. Further experiments should better clarify when the DILP8 size adjustment mechanism is taking place and how systemic ecdysone controls local tissue size assessment to mediate developmental stability. 

The existence of a mechanism in mammals to achieve robust body proportions [60] opens the question of whether the DILP8/Lgr3 homeostatic mechanism is also conserved in mammals. *Drosophila* Lgr3 forms part of a highly conserved subgroup of G protein-coupled receptors (GPCRs) for insulin/relaxin like peptides in metazoans [58,64]. In humans, four relaxin family peptides receptors (RXFP1-4) are described [65]. RXFP1 and RXFP2 show the highest sequence similarity with *Drosophila* Lgr3 [66,67], and both are expressed in the brain [65]. RXFP3, which is slightly different in sequence, has been recently linked to the hypothalamic-pituitary-gonadal axis in rats presumably to control reproduction in the adult [68,69]. However, a function in pubertal development or growth control for the relaxin system is currently unknown. More research is required to determine whether a relaxin dependent mechanism to maintain tissue homeostasis is present in higher organisms.

## 6. AstA/KISS System Coordinates Growth and Maturation in *Drosophila*

We have recently identified another signaling pathway essential for the coordination between growth rate and the growth period [70]. We demonstrated that the AstA/AstAR1 signaling pathway helps to coordinate larval growth with the onset of maturation [70] (Figure 2). By performing a genetic screen to find the upstream PTTHn signals, we have identified AstAR1 as a positive regulator of PTTH neurons. Surprisingly, by combining antibodies and reporter lines, we found that AstAR1 is not only expressed in PTTH neurons but also in the IPCs. Silencing AstAR1 in PTTH neurons resulted in delayed onset of metamorphosis and larger pupae, similar to the phenotype observed in *ptth* null mutants [70]. Conversely, knockdown of AstAR1 in the IPCs reduced larval growth rate resulting in small pupae with unaffected developmental timing, similar to the phenotype we previously reported when silencing *mth* in IPCs [30]. We identified the ligand source for AstAR1, Allatostatin-A (AstA) neuropeptide, which comes from a group of AstA-positive neurons located in the basolateral protocerebrum area. Axon tracing and GFP reconstitution across synaptic partners (GRASP) technique confirmed the existence of physical interaction between the axons of the AstA neurons and the dendrites of PTTH neurons and IPCs. Furthermore, knockdown of AstA in the brain exhibits a combination phenotype of those exhibited by individual loss of AstAR1 in PTTH neurons and the IPCs [70]. Indeed, silencing brain AstA delays the onset of maturation, extending the growth period. However, no pupal overgrowth was observed, indicating that its growth-promoting function is also impaired. Therefore, the AstA signaling pathway coordinates growth with maturation timing to preserve the final target size [70]. However, the main question remains unanswered: What activates/awakes AstA neurons at premetamorphic time to serve as a gatekeeper of metamorphosis?” and thereby “What triggers the metamorphosis at the right time?”

Previous phylogeny studies suggested that AstAR1, Galanin Receptor 1 (GALR1), and Kisspeptin1 Receptor (KISS1R) are evolutionarily related [71,72,73,74,75,76]. Remarkably, the Kiss system is a central modulator of the hypothalamic-pituitary-gonadal axis in mammals. Kiss neurons innervate GnRH neurons and generate the pulsatile GnRH release that induces the onset of puberty [77,78,79]. Indeed, animals that lack either Kiss1 or KISS1 receptor (KISS1R) exhibit prepubertal developmental arrest. Moreover, exogenously administered KISS1 is sufficient to stimulate GnRH secretion, suggesting that KISS1 instructs the onset of puberty [78]. Our findings in *Drosophila* share common aspects with a few major characteristics of mammalian KISS regulation in puberty [2,3,11,70]. For instance, KISS and AstA expression rise during prepubertal or premetamorphic periods. By the same period, GnRH and PTTH neurons became more responsive to KISS and AstA signaling, respectively. In flies, this is reflected by an increased *astAR1* expression. Moreover, KISS and AstA neurons are distributed in two subpopulations. More research, however, is needed to determine whether these neuronal groups are somehow related. Taken together, these data suggest that AstA/KISS system is part of a conserved evolutionary mechanism governing developmental maturation between mammals and *Drosophila* and makes flies a powerful model for identifying signals controlling juvenile to adult transition potentially conserved in higher-order organisms. 

## 7. Conclusions

Although there is a robust understanding of growth and maturation mechanisms, still much needs to be learned about their coordination. Herein, we reviewed the three main *Drosophila* coordinator mechanisms described to date (Figure 2)—growth and steroid hormone feedback, DILP8/GCL, and AstA/AstAR1 coordinator signaling pathways—that might be potentially conserved in mammals. Indeed, in humans, sex steroids also show a differential function in growth regulation. During the juvenile growth period, sex steroids regulate GH secretion to later synchronized systemic growth arrest by restraining GH responsiveness through centralized liver regulations [80]. Moreover, the fact that AstA/KISS1 is an evolutionarily conserved mechanism governing maturation opens the possibility that mammalian KISS1 might concomitantly promote growth with maturation as AstA does [70]. However, the effect of KISS signaling in growth control has been investigated with a lot of controversial results [81,82,83,84,85,86,87]. Consequently, the existence of the KISS coordinator mechanism seems less promising and requires more investigations. Besides, DILP8/LCG signaling toward maturation timing circuitry involved signaling proteins member of the relaxin family, which is conserved in mammals. Therefore, the conservation of this relaxin-dependent surveillance mechanism in higher organisms is an attractive possibility that needs exploration.
